# International public health research involving interpreters: a case study from Bangladesh

**DOI:** 10.1186/1471-2458-5-71

**Published:** 2005-06-28

**Authors:** Emma Pitchforth, Edwin van Teijlingen

**Affiliations:** 1 Department of Health Sciences, University of Leicester, 22–28 Princess Road West, Leicester, LE1 6TP, UK; 2 Edwin van Teijlingen, Department of Public Health and Dugald Baird Centre for Research on Women's Health, University of Aberdeen, Medical School, AB25 2ZD, UK

## Abstract

**Background:**

Cross-cultural and international research are important components of public health research, but the challenges of language barriers and working with interpreters are often overlooked, particularly in the case of qualitative research.

**Methods:**

A case-study approach was used to explore experiences of working with an interpreter in Bangladesh as part of a research project investigating women's experiences of emergency obstetric care.

**The case study:**

Data from the researcher's field notes provided evidence of experiences in working with an interpreter and show how the model of interviewing was adapted over time to give a more active role to the interpreter. The advantages of a more active role were increased rapport and "flow" in interviews. The disadvantages included reduced control from the researcher's perspective. Some tensions between the researcher and interpreter remained hard to overcome, irrespective of the model used. Independent transcription and translation of the interviews also raised questions around accuracy in translation.

**Conclusion:**

The issues examined in this case study have broader implications for public health research. Further work is needed in three areas: 1) developing effective relationships with interpreters; 2) the impact of the interpreter on the research process; and 3) the accuracy of the translation and level of analysis needed in any specific public health research. Finally, this paper highlights the importance to authors of reflecting on the potential impact of translation and interpretation on the research process when disseminating their research.

## Background

Cross-cultural and international research can be illuminating in the public health field, but some important aspects of conducting such research remain under-evaluated. Issues of research practice relating to language are particularly important.[1] To date, much attention has been focused on the conduct of quantitative survey-type research, including the adaptation of questionnaires to achieve equivalence when translating generic health-related measures from one language into another.[1,2] Gaining conceptual equivalence or comparability of meaning in questionnaires is difficult, especially when collecting data in one language and reporting in another.[1,2] Problems of language and cultural translation have real impacts on research outcomes. For example, a review of prevalence data in UK surveys on tobacco and alcohol in ethnic minority groups showed that inadequate cross-cultural adaptation was a potential explanation for discrepancies in reported prevalence.[1]

Conducting qualitative research raises distinctive issues. Unlike questionnaire-based surveys, it may be much more difficult to plan precisely what is going to be said and how. This is partly the case because the interview is a dialogue and certain comments made by interviewees will lead the interviewer to ask different follow-up questions to some interviewees than to others. It is also partly due to the fact that "stories being narrated are constructed in the moments of the interview to the extent that neither the interviewers nor the interviewees can predict the details of what is going to be discussed in advance" (Nunkoosing 2005: 703).[1]

Qualitative research in cross-cultural contexts often relies on interpreters. While there has been a general call to make decisions regarding the use of interpreters and translation in research more explicit using interpreters in public health research is under-researched. [4,8] Several recommendations do exist in other fields and two suggestions are (a) that clarification of roles between the researcher and interpreter before the interview can help to avoid potential problems; [9] and (b) that co-operative working is more likely to be achieved where the researcher appreciates the interpreter's role as actively participative. [10]

This paper examines experiences of working with a lay interpreter during public health qualitative research in Bangladesh. Using a case-study approach, it presents two possible models for working with interpreters used in the study and considers the impact of translation on both the research process and findings.

## Methods

A case study is a research methodology that focuses on the circumstances, dynamics and complexity of a single case or small number of cases.[11] This case study considers the dynamics in cross-cultural research involving an interpreter in addition to the researcher and participants. A case study approach allows detailed examination of the research process and aspects of research practice. [12] An important role for case studies can be the revelation of phenomena that would otherwise be cut off from those at which the work is aimed,[13] and in this instance, involves important consideration of the effects of using an interpreter on the research process and findings.

## Case study

Ethical approval was gained from the Bangladesh Medical Research Council for our research project investigating experiences of women utilizing emergency obstetric care at a large teaching hospital. The research involved a questionnaire administered orally with women as they were admitted to hospital and a more in-depth interview at their home following discharge. The focus of this case study is on the post-discharge interviews; the purpose of this paper is not to present the findings from these interviews, but to examine in detail the methods and effects of working with an interpreter on the research process.

The aim of the interviews was to gather details of the women's 'journey of care' from the decision to go to hospital through to approximately four weeks after discharge. Women and their families were asked in particular about organisational issues, costs incurred and raising the finances required for their treatment episode. Such organisational issues were often the responsibility of family members or neighbours and these people were identified and invited to participate in the interview with the woman who had undergone hospital treatment.

The researcher (EP) had limited understanding of Bangla and employed a lay interpreter for the data collection. The interpreter worked with EP on a full-time basis for a period of six months. The background characteristics of the researcher, interpreter and main participants are presented in Table 1.

**Table 1:** Characteristics of researcher, interpreter and participants

**The researcher**	**The interpreter**	**The participants**
Female	Female	Female
Aged 25	Aged 25	Aged 15–45
Unmarried	Unmarried	Married
No children	No children	Children
Ph.D. student	Master's education	Limited formal education
Non-poor	Non-poor	Poor
Christian	Muslim	Muslim/Hindu
Scottish	Bangladeshi	Bangladeshi
No experience working with interpreter	No experience working as interpreter	Unknown experience of research

The interpreter was identified through a research and evaluation unit at a large non-government organisation in Bangladesh. She had excellent spoken and written English but was not trained as an interpreter, although she did have experience of research interviewing and had a health-related postgraduate qualification. The researcher and the interpreter spent considerable time discussing how best to carry out the interviews. Following previous advice, this included discussion over the respective roles of the interpreter and researcher. For example, the interpreter was asked to interpret all of the participants' answers and the researcher's questions. The researcher stressed that it was important to hear participants' responses even if the question or anticipated answer seemed obvious to the interpreter. The interview schedule was devised and written in English and time was spent ensuring that it would be culturally acceptable to the interviewees.

## Interview process

**Figure 1:**
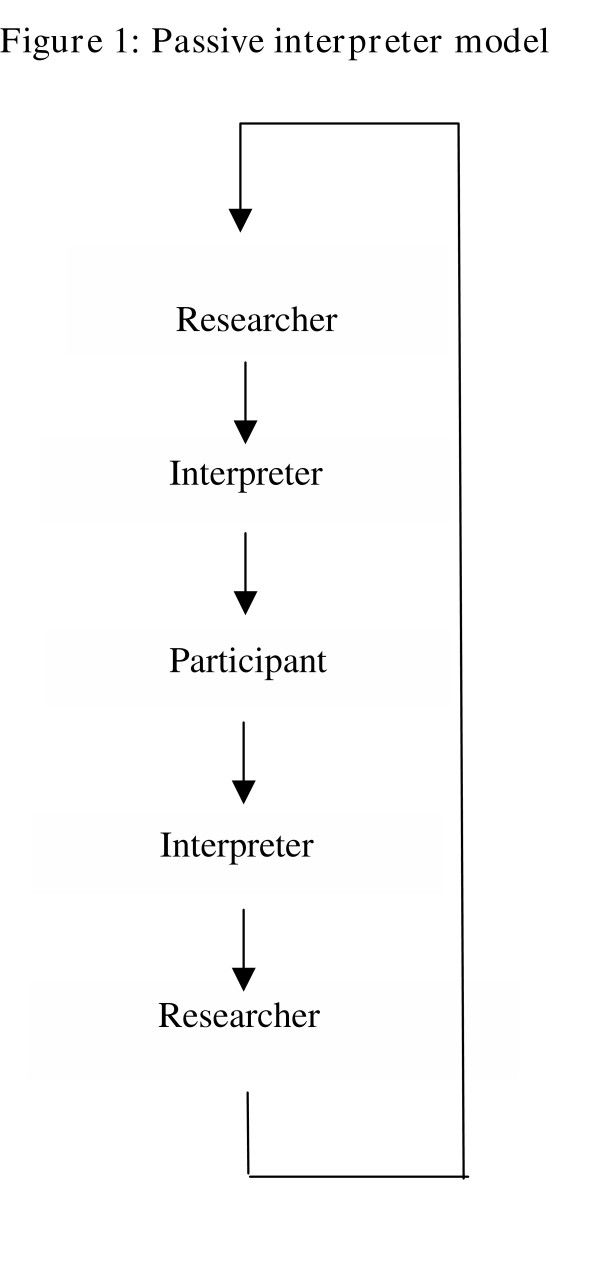
Passive interpreter model.

Figure 1 illustrates the model of interviewing that was adopted for the initial interviews. This reflects a passive model, in which the researcher asked questions through the interpreter, who would then interpret the response from the participant to the researcher. This method was adopted because it enabled the researcher to follow the entire interview and to ask further questions, as required.

During the interviews it became clear that there were a number of disadvantages to this approach. The interpretation back and forth made the interviews time consuming. The interviews often brought the women and their families away from other work and this model of interviewing seemed to reduce the focus on the participants, as they had to wait while information was interpreted. This model also involved some tensions between the researcher and the interpreter, including occasions when the interpreter did not interpret either the question to the participant or the answer back to the researcher. There were other such occasions when the interpreter clearly felt a question was obvious or did not want to ask it and would sigh loudly or raise her eyebrows. A benefit of qualitative interviewing is that the interviews can flow like a conversation rather than a structured question and answer situation – a guided conversation with a purpose.[14] This flow was restricted as the interpreting interrupted the dialogue between participants and the interpreter and in practice the interviews often became disjointed.

**Figure 2:**
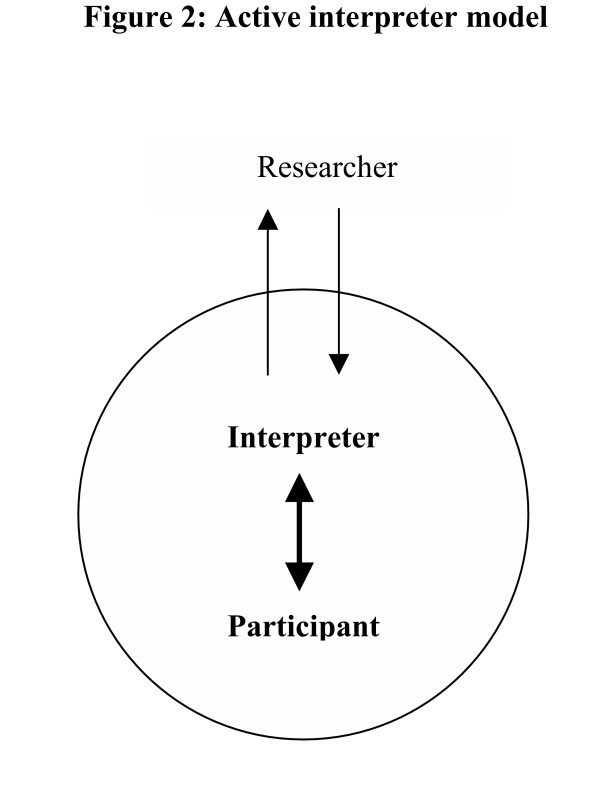
Active interpreter model.

The researcher and interpreter reflected on the progress of the interviews and it was felt that a new model of interviewing should be adopted. The interviews were semi- structured and the interpreter had quickly become familiar with the aims of the interview and interview schedule. The model of interviewing was subsequently changed to an active model, so that the interpreter carried out most of the interview (Figure 2), with the aim of improving the flow of interviews and making the interviews less burdensome. The researcher was always present and at key points the interpreter would summarise the interview to the researcher to allow additional questions to be asked. With the participants' permission, all of the interviews were audio-taped and transcribed so that the full interview transcript was available to the researcher.

This second model did allow interviews to flow more like a conversation, enabling the interpreter to build a better rapport with participants and reduced the time burden for participants. Allowing the interpreter to take more control in the interview also reduced some of the tensions inherent in the first model, but it did so by reducing the control and involvement of the researcher (situated outside the circle in Figure 2). Of course, in either model the researcher has reduced control compared to direct interviewing between researcher and participant.

Some differences arose irrespective of the model used. For example, the researcher and interpreter differed as to how the role of the researcher-interpreter team should be perceived by the respondents. The researcher felt that it would be important to emphasise that they were not associated with the hospital so that the women and their families felt that they could talk freely about their experiences.[15] It was hoped that the researcher was not associated with any form of authority.[16] The interpreter, however, placed less importance on this, introducing herself as a nutritionist working for a non-government organization, backed up with the name of a well-known doctor. The interpreter also placed less emphasis on introductions and informed consent. She felt that if families did not want to talk to us they would not do so. The researcher and interpreter had, in addition, discussed and (apparently) agreed that it was not appropriate to be judgemental about decisions made by families. However the researcher, with growing understanding of Bangla, was aware of occasions when the interpreter would take issue with the participants, e.g. when a woman reported that she had not attended antenatal check-ups during her pregnancy.

## Transcribing, interpreting and translating

With permission, all of the interviews were audio taped and transcribed in English. This was done by the same interpreter. A thematic analysis of the transcripts was planned and as a quality-control measure to check accuracy and validity, four interviews were transcribed by a bilingual interpreter in the UK. Comparisons were to be made within and across themes and to show common and differing experiences among women.[17,18]

The transcripts from the original and independent translations were compared and contrasted. Some differences were noted but overall the researchers were reassured that they were similar enough for the purpose of this public health study. Any differences tended to be in over-interpretation of women's own words, level of precision and emphasis.

The extract below shows, for example, that there may have been a tendency on the part of the lay interpreter to "interpret" the women's words rather than directly translate. In the original transcripts the word "forceps" is used but in the independent translation, the description of a metal cup on the baby's head is more likely to have been the words used by the woman:

"There the doctors also tried but they failed to get my delivery. They tried to deliver by forceps also" (original translation)

Compared to the less technical:

"The doctors tried to take the baby out by using a metal cup on the baby's head but they were unsuccessful" (independent translation)

There were also occasions when the different transcripts did not vary considerably in content but the emphasis may have been interpreted differently. For example the tone of the doctors is not portrayed in the same way in the following two examples.

"One doctor came to me to say that it is not possible for us, you have to take your patient. After a while the nurses also said the same thing. Then they took me to Dhaka Medical College Hospital" (original translation)

Compare this with the following:

"One doctor said, 'we can't do it here. Take her away!' Later seven nurses tried, but then we won't be able to do it. Then my father took me away" (independent translation)

Similarly, the example below shows the differences in the level of detail included in the transcripts. The interpreter asked:

"Was the ambulance arranged by Red Crescent Hospital or by your own relatives?"

The original translation of the reply was:

"By Red Crescent Hospital" (original translation)

Whilst the independent translation was more subtle:

"At first they told us to make our own way – but after my uncle spoke to them, they arranged to bring me in the ambulance" (independent translation)

However, both excerpts show that the Red Crescent Hospital arranged the ambulance but the detail included in the independent translation gives greater insight into the process involved.

We are very well aware that different academic disciplines might approach the detail of transcribing and/or analysing differently. For example, a student of Psychology might be interested in the 'pauses' and 'hmms' and 'sighs' in the conversation to establish how emotionally challenging the conversation is for the woman, whilst a student of Women's Studies might be interested in the power differences between the female interviewees and their male family members, whilst a student of Management Studies might focus on the way health care professionals manage the woman's situation within their limited resources. In Public Health we are often interested in groups of people rather than individual stories or events. As such, in more applied studies like this one, detailed transcription and some differences in the translations are not necessarily as significant as they may be in other disciplines.

## Discussion

This case study shows some of the challenges that can be faced when conducting qualitative research with an interpreter. The problems identified are not necessarily new, but we would argue that the advice that many of them can be avoided by clarifying the role of the researcher and interpreter might be somewhat naïve. For example, it has been suggested that the respective roles of the researcher and interpreter and rules of research governance could be established before the interview. In practice, however, we found the reduced control inherent in relying on an interpreter meant that the agreed procedure was not always followed. Using the interpreter in a traditional passive role introduced tensions into the research interviews and was burdensome for the participants. The alternative 'active-interpreter' model that was adopted gave the impression of overcoming these tensions but did so by effectively excluding the researcher for part of the interview. Again, the view that the researcher can avoid such problems by seeing the interpreter's role as actively participative seemed difficult to implement.

This paper has used a case study approach to illustrate the issues involved in using an interpreter in cross-cultural research. Common criticisms of case studies are that they provide little basis for scientific generalization and that there can be difficulty in assessing the importance of relationships, which may be simply idiosyncratic to one particular case.[18,20] The nature of case studies, however, means that the extrapolation of findings does not depend on the representativeness of the case, but on the clarity of the theoretical reasoning.[21] The generalisability of the findings in this case study would need to be established; it is likely that the nature and context of the research and interviews would have important effects. That said, case studies are well suited to areas where previous research is lacking and in this case highlights that more practical advice is needed on developing effective relationships between the researcher-interpreter team.

In this respect our case study demonstrates vividly the importance of reflexivity in conducting this type of research. Reflexivity is widely encouraged within qualitative research as a means for researchers to reflect upon, critically examine and analytically explore the nature of the research to demonstrate assumptions about gender or other relationships which are built into the research.[22] The process of translation and use of interpreters is an important part of such reflexive methodology.[10] It can be argued that research involving an interpreter can add additional layers of bias within the interview process.[23] Jentsch showed diagrammatically how the background characteristics, psychological factors and behavioural factors of the researcher, interpreter and respondents may interact.[9] The background characteristics and behaviour may have an impact on psychological factors (such as attitude and expectations), which in turn influence behaviour.

Taking the background characteristics of all three parties in our case study (Table 1), there are clear differences but also some similarities. First and foremost, all were the same gender. To do research in a traditional society such as Bangladesh requires cultural sensitivity from the part of the researcher and research on such as female oriented topic also requires a female researcher and interpreter. [24] The interpreter shared some characteristics with the researcher and some with the respondents. An advantage of using an interpreter, born and brought up in Bangladesh, was that she was able to act as a 'cultural interpreter', giving a greater insight into the interviews with the women and Bangladesh more generally. The respondents could also relate immediately to the interpreter which helped facilitate the interviews.[9]

Issues of sameness and difference have been highlighted in the literature by drawing on the influence of 'insider' and 'outsider' status.[25-27] Gender, racial identity, social class and shared experience can affect the research process and willingness of respondents to talk to the researchers.[25] It has been argued that the relationship between interviewer and participant should be non-hierarchical to best understand their life experiences. [28] The simple comparison of background characteristics in our case study shows that, aside from gender, the researcher could definitely be considered an 'outsider' but this need not be detrimental to the research and it was felt that the differences between all three parties may have been beneficial. The obvious differences in background characteristics such as wealth and educational attainment gave opportunity for those involved to articulate their feelings about their different life experiences.[29]

In addition to the effect of an interpreter on the research process it is important to make explicit decisions about the use of translations and presentation of findings. The use of only one interpreter was seen as advantageous as it helps ensure reliability in translation.[30] The independent translation of a small number of interviews was then used to assess validity.[30,31] The case study has highlighted the differences rather than similarities between the transcripts and, overall, it was judged that, for this research project, the differences were not sufficiently significant to impact on the thematic analysis undertaken. In contrast, it could have raised significant problems had our study been to analyse (1) how women describe the decision-making process, for example, around transfer to hospital; or (2) the lay understanding of maternity service provisions. The differences in language that were highlighted did not effect our interpretation of the findings. Interestingly, they suggested that the original interpreter was more likely to medicalise terms and was less detailed. It is not that one translation should be considered right or wrong, but the value is in appreciating differences in interpretation in order to discuss different possible perspectives on the research findings.[31]

Finally, it is recognised in medical sociology that differences in interpretation between researcher and interpreter are not simply differences in translation, but are part of a wider phenomenon, namely that concepts of health and illness are socially constructed. [32-34] In other words, a particular woman in Bangladesh having certain obstetric complications is not a taken-for-granted fact based on medical scientific evidence, but it is a renegotiation of medical knowledge within a cultural and social context. This has an impact on the disease under debate, but also in the way one deals with it in terms of health care provision.[35]

## Conclusion

Issues of cross-language data collection should be seen as a challenge and not as an obstacle. [1] This paper presents two different models of practice for working with an interpreter. The 'passive-interpreter' and 'active-interpreter' model each had advantages and disadvantages as discussed. Despite efforts to clarify roles between the researcher and interpreter some areas of tension were hard to overcome. Greater attention should be paid to the effect of an interpreter on the research process. As Temple and Young reminded us: "The translator always makes her mark on the research, whether this is acknowledged or not…".[36] Developments should include more practical advice to enable researchers and interpreters to develop more effective relationships.

In terms of the research findings this study highlights the importance of considering the relationship between researcher, interpreter and respondents and the inclusion in reflexive methodology. The challenges faced when using translation in qualitative research will depend upon the level of depth in analysis and accuracy in translation required. For some studies a less accurate, but perhaps easier to read translation will be sufficient. This should be considered at the outset of study design so that the level of accuracy achieved does not compromise the level of analysis undertaken. The type of analysis planned and study design should drive the level of accuracy in translation rather the level in accuracy limiting the possible analysis.

In conclusion, we would like to recommend that public health researchers working with interpreters and translations pay more attention to: 1) developing effective relationship with interpreter; 2) the effect of the interpreter on the research process; and 3) the accuracy of the translation and level of analysis needed any specific public health research.

## Competing interests

The author(s) declare that they have no competing interests.

## Author's contributions

EP carried out the data collection and analysis for this study as part of her Ph.D. research. EvT was joint supervisor of this public health research project and participated at all stages of the study. Both authors have written several drafts and approved the final manuscript.

## Pre-publication history

The pre-publication history for this paper can be accessed here:


